# Efficacy and toxicity of Iodine-125 seed implantation for lymph node recurrence secondary to esophageal cancer after radiotherapy: a multicenter retrospective study

**DOI:** 10.1186/s13014-022-02196-y

**Published:** 2023-01-26

**Authors:** Lirong Wu, Xinxin Zhao, Suqing Tian, Kaixian Zhang, Chuang He, Yong Feng, Jiawei Zhou, Wenjie Guo, Zhe Ji, Xia He, Guanglie Chen, Junjie Wang

**Affiliations:** 1 Department of Radiation Oncology, Jiangsu Cancer Hospital, Jiangsu Institute of Cancer Research, The Affiliated Cancer Hospital of Nanjing Medical University, Nanjing, 210009 China; 2 Department of Radiation Oncology, The First People’s Hospital of Kerqin District, No. 328 Kerqin Street, Tongliao, 028000 China; 3 Department of Radiation Oncology, Beijing University Third Hospital, Beijing, 100191 China; 4 Department of Oncology, Tengzhou Central People’s Hospital Affiliated to Jining Medical College, Tengzhou, 277599 China; 5 Center of Minimally Invasive Intervention, Southwest Hospital of Army Medical University (The First Hospital Affiliated to AMU), Chongqin, 400038 China

**Keywords:** Radioactive Iodine-125 seed, Esophageal cancer, Radiotherapy, Prognosis, Toxicity

## Abstract

**Background/objective:**

This multicenter study aimed to explore the efficacy and toxicity of radioactive Iodine-125 seed implantation for lymph node recurrence in patients with esophageal cancer after external radiotherapy.

**Methods:**

Clinical data of eligible patients from 5 centers in China were retrospectively reviewed. A total of 126 patients between January 2016 and March 2019 were included. The median interval between previous radiotherapy and radioactive Iodine-125 seed implantation was calculated. The target volume was 2.1–128.1 cm^3^ (median, 22.2 cm^3^) and the median postoperative D90 is 120.6 Gy (range, 101.7–192). Short-term efficacy of tumor response, the long-term efficacy of local progression-free survival (LRFS) and overall survival (OS), and treatment-related toxicity were reported.

**Results:**

For tumor response, 37 (29.4%), 51 (40.5%), 14 (11.1%), and 24 (19.0%) patients achieved complete response, partial response, stable disease and progressive disease, respectively. The 1-, 2- and 3-year LPFS and OS rates were 48.8%, 23.0% and 15.9%, and 80.2%, 38.8%, and 24.5%, respectively. Multivariate analysis identified Karnofsky performance status (*P* = 0.041) and tumor response (*P* = 0.049) as independent prognostic factors for LPFS; initial tumor stage (*P* = 0.034), lesion volume (*P* = 0.017), and tumor response (*P* = 0.004) as independent prognostic factors for OS. In total, 77 (61.1%) patients suffered from skin reactions and the incidence of grade 3–5 skin toxicity was 5.6% (7/126).

**Conclusion:**

Radioactive Iodine-125 seed implantation seems efficient with acceptable toxicity for the treatment of lymph node recurrence secondary to esophageal cancer. A head-to-head study is needed to further evaluate the survival benefit.

## Background

As the seventh most frequently diagnosed cancer and the sixth leading cause of cancer-related deaths worldwide [1], esophageal cancer (EC) has a very poor prognosis with a 5-year overall survival (OS) of 20% [2]. Patients with early-stage disease are treated by radical surgery while concurrent chemoradiotherapy (CRT) remains the standard care for patients with advanced disease unsuitable for surgery [3]. Although advances in radiotherapy techniques and chemotherapy regimens have been achieved in recent years, the prognosis of advanced-stage cancers remains unsatisfactory and more than 50% of patients would finally experience disease recurrence, with a 3-year OS less than 56% [4–10]. Unfortunately, effective treatment strategies are lacking for patients experiencing recurrence and needed further investigation.

In recent years, there is increasing data showing that radioactive Iodine-125 seed implantation is an effective and safe treatment for various malignant diseases [11–13]. The main advantage of seed implantation is that a higher dose could be achieved in the tumor volume while the dose to surrounding normal tissues is low [13–17]. Particularly, radioactive Iodine-125 seed implantation has become the first-line treatment for early-stage prostate cancer.

With the introduction of computed tomography (CT)-guided technology, three-dimensional printing coplanar template (3D-PCT), and 3D printing noncoplanar template (3D-PNCT), the accuracy of radioactive Iodine-125 seed implantation has been greatly improved, broadening its clinical application. However, little is known about the efficacy of radioactive Iodine-125 seed implantation for lymph node recurrence secondary to EC after radiotherapy in a multi-center setting. Therefore, this retrospective multicenter study aims to evaluate the efficacy and toxicity of radioactive Iodine-125 seed implantation for lymph node recurrence secondary to EC after radiotherapy.

## Materials and methods

### Study patient

This study was approved by the ethics committee of the five centers and performed following the declaration of Helsinki. We retrospectively reviewed the clinical data of patients with EC between January 2016 and March 2019 at 5 centers in China (Jiangsu Cancer Hospital, Tengzhou Center Hospital, Tongliao Hospital, Southwest Hospital, and Peking University Third Hospital). Each enrolled patient underwent a multidisciplinary discussion before treatment. Patients meeting the following criteria were eligible for this study: (1) received radioactive Iodine-125 seed implantation for lymph node recurrence secondary to EC; (2) previously received radiotherapy or CRT. The indications for radioactive Iodine-125 seed implantation in our study were similar to those described previously [13] and were all as follow: (1) Age ≥ 18 years; (2) Pathological or CT/MRI/Ultrasound imaging confirmed lymph node recurrence after radiotherapy; (3) Failed or not eligible to surgery, chemotherapy, and radiotherapy or patients refused these treatments, and no other anti-cancer treatments available; (4) Greatest diameter of lymph node recurrence < 7 cm; (5) No bleeding tendency and without oral aspirin/anticoagulant drug; (6) Good physical status (KPS > 70) and expected survival > 1 year; (7) Curative intent for lymph node recurrence only or palliative intent for symptomatic lymph node recurrences, such as pain, numb and edema. Written informed consent was obtained from patients before treatment.

### Radioactive Iodine-125 seed implantation

Firstly, patients received an enhanced CT scan (thickness, 5 mm) before the treatment and the images were loaded into the treatment planning system to assess the feasibility of radioactive Iodine-125 seed implantation. The preoperative plan would be designed based on the CT images, including direction, distribution, and depth of seed needles, gross tumor volume, seed amount, and activity. Then, seed needles were inserted into the targeted lesion under CT guidance after local anesthesia with 1% lidocaine. According to the preoperative plan, the needle should be at least 0.5 cm from the tumor edge and the distance between needles should be 0.5 to 1.0 cm. The seeds were implanted using a mick seed implantation gun during the process of withdrawing the gun, leaving a distance of 0.5–1.0 cm between seeds. Subsequently, a CT scan would be performed to check the distribution of the seeds. A course of antibiotics and hemostatic would be given to patients to prevent infection and bleeding.

### Efficacy assessment

Short-term efficacy evaluation is tumor response according to the Response Evaluation Criteria in Solid Tumors (version 1.1) [18] by two radiologists separately, and consensus for conflicting settings, based on CT scan performed three months after implantation. Only lymph node recurrence that received radioactive Iodine-125 seed implantation were included in the tumor response evaluation. Complete response (CR) was defined as disappearance of the target lesion, partial response (PR) as at least 30% reduction of target lesion volume from baseline, progressive disease (PD) as at least 20% increase of target lesion volume, and stable disease (SD) as between PR and PD.

Patients were followed every 3 months after radioactive Iodine-125 seed implantation by CT scan. Long-term efficacy in our study included overall survival (OS, defined as the time interval between radioactive Iodine-125 seed implantation and death from any cause) and local progression-free survival (LPFS, defined as the time interval between radioactive Iodine-125 seed implantation and the progression of recurrent lymph nodes which received radioactive Iodine-125 seed implantation). Notably, other progressive events such as local–regional recurrence, distant metastasis, and the progression of lymph nodes that did not receive radioactive Iodine-125 seed implantation were not included in LPFS analysis.

### Adverse events

Toxicities of radioactive Iodine-125 seed implantation were recorded and graded according to the Common Terminology Criteria for Adverse Events (version 4.0).

### Statistical method

Differences of categorical between groups were compared using Chi-square or Fisher’s exact test, and t-test was employed for the comparisons between continuous variables. Kaplan–Meier method was used to establish the estimated survival outcomes and differences between groups were compared by Log-rank test. Multivariate analysis using Cox proportional hazard model was applied to establish independent prognostic factors and their corresponding hazard ratios (HRs) and 95% confidence intervals (CIs). All tests were two-sided and *P* < 0.05 was considered significant and Stata Statistical Package 12 (StataCorp LP, College Station, TX, USA) was used for all analyses.

## Results

### Patient characteristics

Between January 2016 and March 2019, a total of 126 eligible patients treated at five centers were included in our study and their baseline information were shown in Table 1. Among the whole cohort, 114 (88.9%) were male and 12 (11.1%) were female, aged 47 to 80 years. Most of patients (124/126, 98.4%) had a KPS ≥ 80 and only patient had adenocarcinoma disease (0.8%). A total of 116 (91.1%) patients received previously adjuvant CRT or radiotherapy alone, and the other patients received radiotherapy for recurrent disease. The external radiation dose ranged from 48 to 60 Gy and the median time interval between radioactive 125-I seed implantation and last radiotherapy is 13 months (range, 8–30 months). For lymph node recurrence, only two patients (1.5%) experienced multiple lesions involvement. Moreover, 86 patients had clinical symptoms associated with recurrent lymph nodes including regional pain (n = 79, 62.7%) and numb (n = 7, 5.6%). Additionally, 39 (31.0%) patients also suffered local disease progression.

**Table 1:** Baseline information of the 126 patients

Characteristics	Number	Percentage (%)
Gender		
Male	112	88.9
Female	14	11.1
Median age (range)	63 (47–80)	
Karnofsky performance status		
70	2	1.6
80	50	39.7
90	74	58.7
Pathology		
Squamous	125	99.2
Adenocarcinoma	1	0.8
Initial T category^a^		
T1	10	7.9
T2	19	15.1
T3	88	69.9
T4	9	7.1
Initial N category^a^		
N0	37	29.4
N1	62	49.2
N2	18	14.3
N3	9	7.1
Initial overall stage^a^		
I	6	4.7
II	39	31.0
III	63	50.0
IVA	18	14.3
Initial treatment		
Surgery + adjuvant CRT	67	53.2
Radical CRT	40	31.7
Surgery + adjuvant RT	9	7.1
Surgery + adjuvant Chemotherapy	5	4.0
Surgery alone	5	4.0
The boundary of recurrent lymph node		
Clear	95	75.4
Non-clear	31	24.6
Number of recurrent lymph node		
Single	48	38.1
Multiple	78	61.9
Lesion of recurrent lymph node		
Cervical only	37	29.4
Supraclavicular only	64	50.8
Mediastinum only	23	18.3
Two or more lesions	2	1.5
Recurrent lymph node-related symptoms		
Pain	79	62.7
Number	7	5.6
Local disease		
Under control	87	69.0
Progression	39	31.0

*CRT* concurrent chemoradiotherapy, *RT* radiotherapy

^a^According to the 8th edition of the UICC/AJCC staging workup

### Procedure details of Iodine-125 seed implantation

All radioactive Iodine-125 seeds were implanted at the site of recurrent lymph nodes. The information on radioactive Iodine-125 seed implantation were shown in Table 2. In detail, 68 (54.0%) and 58 (46.0%) patients received CT guided implantation and 3D-PNCT implantation, respectively. The target volume was 2.1–128.1 cm^3^ (median, 22.2 cm^3^) and the prescribed dose was 100–180 Gy (median, 120 Gy). The seed activity ranged from 0.38 to 0.8 mCi (median, 0.6), the seed number was 7–120 (median, 43) and a median of 10 needles (range, 1–38) were used to achieve such implantation. Moreover, the median post-operative D90 is 120.6 Gy (range, 101.7–192).

**Table 2:** Information of radioactive ^125^I seed implantation for the 126 patients

Characteristics	Median value	Range
Target lesion volume (cm^3^)	22.2	2.1–128.1
Prescribed dose (Gy)	120	100–180
Seed activity (mCi)	0.6	0.38–0.8
Seed number	41	7–120
Needle number	10	1–38
D90 (Gy)	130.3	101.7–192

### Efficacy

**Fig. 1:**
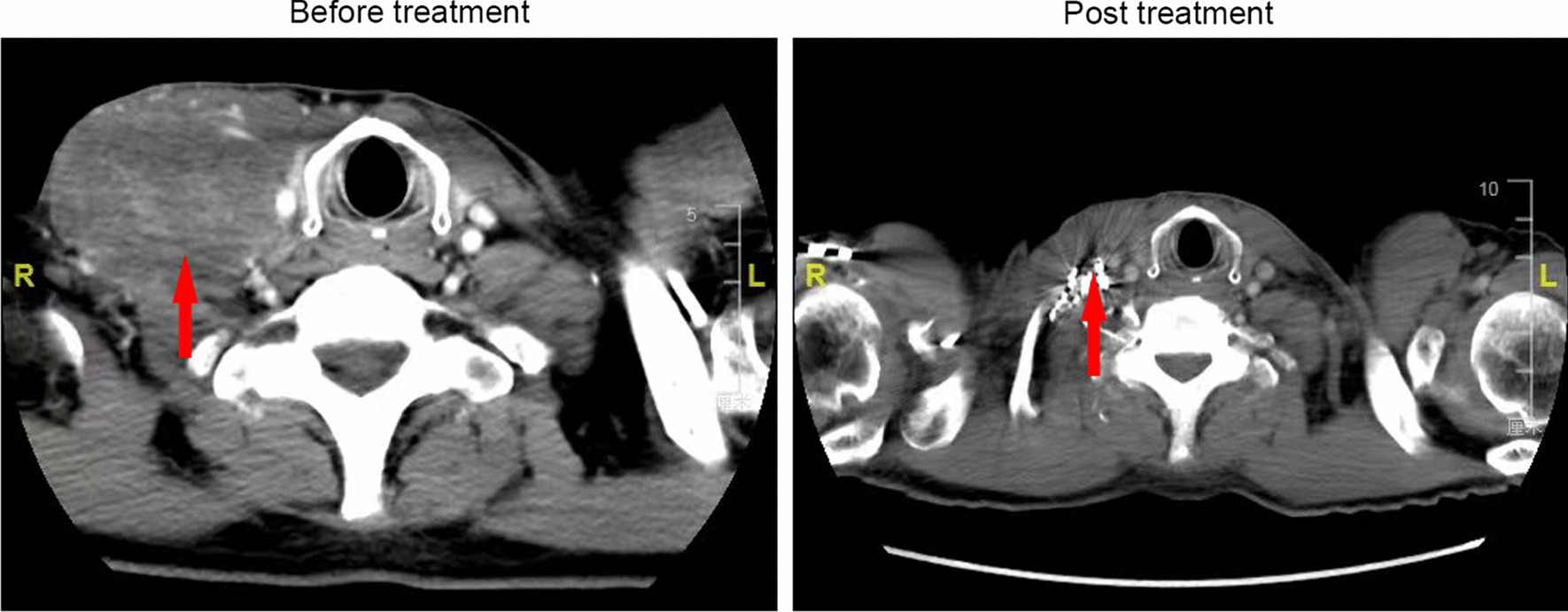
Recurrent neck lymph node before and post Iodine-125 seed implantation for patient with complete response

Three months after radioactive Iodine-125 seed implantation, 37 (29.4%) patients had CR (Fig. 1), 51 (40.5%) patients had PR, 14 (11.1%) patients had SD, and 24 (19.0%) patients suffered from PD, achieving a disease control rate (CR + PR + SD) of 81% (102/126). Of the 86 patients with clinical symptoms, 53 (61.6%) of them experienced relief of regional pain and numbness.

**Fig. 2:**
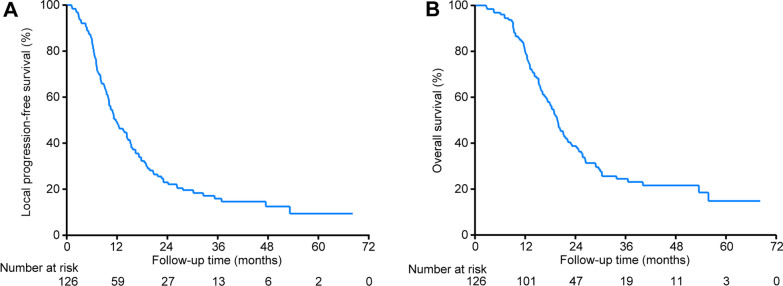
Kaplan–Meier local progression-free survival (**A**) and overall survival (**B**) curves for the whole cohort

Up to the last follow-up (September 2021), the median follow-up duration for the entire cohort was 19.57 months (range, 2.7–68.17 months). Finally, 105 (83.3%) patients experienced lymph node progression and 97 (77.0%) patients died. The main cause of death was disease progression and only 7 (7.2%) deaths were due to non-cancer-related reasons including pulmonary infection (n = 2), massive hemorrhage from skin ulcer (n = 1), tracheoesophageal fistula (n = 3), and accident (n = 1). The median LPFS and OS were 11.0 months (range, 1.2–68.17 months) and 19.57 months (range, 2.7–68.17 months), respectively. The 1-, 2- and 3-year LPFS and OS rates were 48.8%, 23.0% and 15.9% (Fig. 2A), and 80.2%, 38.8% and 24.5% (Fig. 2B).

### Prognostic factors analysis

**Fig. 3:**
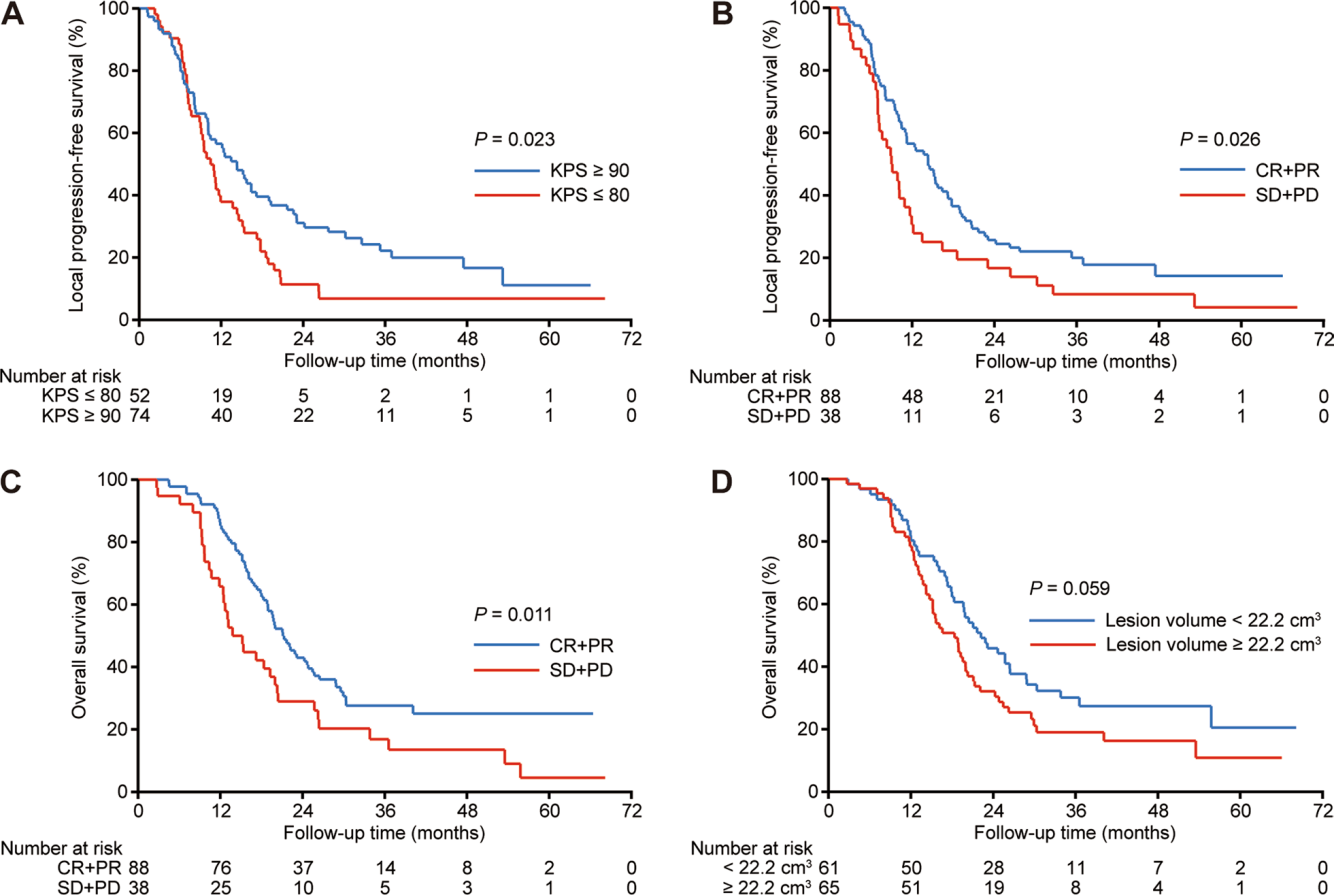
Kaplan–Meier local progression-free survival (**A** and **B**) and overall survival (**C** and **D**) between different subgroups

We performed subgroup analysis to identify potential prognostic factors associated with radioactive Iodine-125 seed implantation (Table 3). Univariate analysis showed that KPS ≥ 90 (*P* = 0.023; Fig. 3A) and CR/PR (*P* = 0.026; Fig. 3B) were associated with better LPFS while only short-term efficacy of CR/PR was associated with better OS (*P* = 0.011; Fig. 3C). Notably, a significant difference was observed between lesion volume < 22.2 cm^3^ and ≥ 22.2 cm^3^ for OS (*P* = 0.059; Fig. 3D). Results of multivariate analysis revealed that KPS (HR, 0.662; 95% CI, 0.446–0.983; *P* = 0.041) and tumor response (HR, 1.513; 95% CI, 1.002–2.284; *P* = 0.049) were independent prognostic factors for LPFS while initial tumor stage (HR, 0.617; 95% CI, 0.395–0.964; *P* = 0.034), lesion volume (HR, 1.679; 95% CI, 1.099–2.566; *P* = 0.017) and tumor response (HR, 1.876; 95% CI, 1.222–2.878; *P* = 0.004) were independent prognostic factors for OS (Table 4).

**Table 3:** Subgroup analysis of prognostic factors by univariate analysis

Factor	Number	LPFS					OS				
		Median (month)	1-year (%)	2-year (%)	3-year (%)	*P* value	Median (month)	1-year (%)	2-year (%)	3-year (%)	*P* value
Age (years)						0.322					0.228
< 63	59	11.23	48.7	26.1	19.9		19.67	81.4	38.9	32.7	
≥ 63	67	11.0	48.9	20.1	12.1		19.9	79.1	38.6	17.2	
KPS						0.023					0.129
≤ 80	52	10.17	37.9	11.4	6.8		18.88	78.8	32.2	15.6	
≥ 90	74	13.0	56.5	31.1	22.2		19.98	81.1	43.2	30.6	
Initial tumor stage						0.296					0.305
I–II	45	10.13	44.0	18.5	9.7		18.37	77.8	31.1	21.8	
III–IVA	81	11.93	51.4	25.5	19.5		19.97	81.5	43.1	25.4	
No. of recurrent lymph nodes						0.795					0.389
Single	48	11.92	51.6	23.6	12.9		19.82	79.2	41.7	31.0	
Multiple	78	10.85	47.1	22.7	17.6		19.52	80.8	36.9	19.9	
Boundary of recurrent lymph nodes						0.565					0.838
Clear	95	11.17	48.8	19.7	14.8		19.97	83.2	37.7	23.9	
Non-clear	31	11.23	48.4	32.3	17.4		16.13	71.0	41.9	24.3	
Lesion volume (cm^3^)						0.165					0.059
< 22.2	61	13.47	55.5	27.8	18.2		22.23	82.0	45.9	30.1	
≥ 22.2	65	10.9	42.6	18.6	13.5		18.37	78.5	32.1	19.0	
Prescribed dose (Gy)						0.6					0.525
≤ 120	70	10.28	45.2	18.1	16.1		19.47	77.1	35.7	22.5	
> 120	56	13.03	53.2	29.1	15.2		20.15	83.9	42.7	27.3	
D90 (Gy)						0.178					0.886
≤ 130.3	62	10.62	51.1	29.7	23.3		19.83	79.0	41.8	25.7	
> 130.3	64	11.45	56.3	17.2	12.5		19.67	81.3	35.9	23.0	
Time interval between RT and Iodine-125 seed implantation (months)						0.42					0.326
≤ 13	67	11.23	47.5	22.7	12.7		18.97	79.1	34.1	22.1	
> 13	59	11.0	50.3	23.4	19.2		21.67	81.4	44.0	27.4	
Seed activity (mCi)						0.561					0.219
≤ 0.6	75	11.23	48.8	26.2	16.3		20.3	86.7	42.4	27.8	
> 0.6	51	11.0	48.8	18.3	15.3		17.23	70.6	33.3	19.7	
Short-term efficacy						0.026					0.011
CR + PR	88	14.32	56.6	25.7	20.0		21.1	86.4	43.0	27.6	
SD + PD	38	9.05	30.7	16.7	8.4		14.52	65.8	28.9	16.9	

*KPS* karnofsky performance status, *LPFS* local progression-free survival, *OS* overall survival, *RT* radiotherapy, *CR* complete response, *PR* partial response, *SD* stable disease, *PD* progressive disease

**Table 4:** Multivariate analysis of prognostic factors

Endpoints	Factor	HR (95% CI)	*P* value
LPFS	KPS (≥ 90 vs. ≤ 80)	0.662 (0.446–0.983)	0.041
	Short-term efficacy (SD + PD vs. CR + PR)	1.513 (1.002–2.284)	0.049
OS	Initial tumor stage (I + II vs. III + IVA)	0.617 (0.395–0.964)	0.034
	Lesion volume (≥ 22.2 vs. < 22.2 cm^3^)	1.679 (1.099–2.566)	0.017
	Short-term efficacy (SD + PD vs. CR + PR)	1.876 (1.222–2.878)	0.004

*P* values were calculated using an adjusted Cox proportional hazard model with the following factors: age (≥ 63 vs. < 63 y), KPS (≥ 90 vs. ≤ 80), initial tumor stage (III + IVA vs. I + II), number of lymph nodes (single vs. multiple), lesion volume (≥ 22.2 vs. < 22.2 cm^3^), prescribed dose (> 120 vs. ≤ 120 Gy), D90 dose (> 130.3 vs. ≤ 130.3 Gy), the time interval between radioactive Iodine-125 seed implantation and previous radiotherapy (> 13 vs. ≤ 13 months), seed activity (> 0.6 vs. ≤ 0.6 mCi), the boundary of recurrent lymph nodes (non-clear vs. clear) and short-term efficacy (SD + PD vs. CR + PR)

*KPS* karnofsky performance status, *CR* complete response, *PR* partial response, *SD* stable disease, *PD* progressive disease, *LPFS* local progression-free survival, *OS* overall survival, *HR* hazard ratio, *CI* confidence interval

### Toxicity

In total, 77 (61.1%) patients suffered from skin toxicities while the incidence of severe skin toxicities (grade 3–5) was only 5.6% (7/126). Of these patients, 52 (67.5%) were grade 1, 18 (23.4%) were grade 2, and 6 (7.8%) were grade 3. The patient with grade 5 suffered from a massive hemorrhage due to a radioactive Iodine-125 seed implantation-related skin ulcer. Generally, grade 1 or more skin toxicity was more frequent in higher D90 dose (> 130.3 vs. ≤ 130.3 Gy: 73.4% vs. 48.4%, *P* = 0.004) while had no significant correlation with prescribed dose (> 120 vs. ≤ 120 Gy: 64.3% vs. 58.6%, *P* = 0.513). No other toxicity was observed.

## Discussion

Radiotherapy plays a pivotal role in the treatment of EC. Patients with advanced EC unsuitable for surgery would receive radical CRT with or without induction chemotherapy [3, 10, 19, 20]. Also, radiotherapy together with chemotherapy as neoadjuvant and adjuvant treatments have been widely applied to improve local control and eradicate micrometastases [21–24]. However, toxicities and physiological changes of normal tissues after radiotherapy usually make surgery or re-irradiation unsuitable or unavailable for recurrent disease. In recent years, radioactive Iodine-125 seed implantation has been increasingly applied for clinical cancer treatment, especially for those with recurrent disease after radiotherapy, as a result of its low energy, rapid dose decrease with distance, and minimally invasive nature [25, 26]. Radioactive Iodine-125 seed could deliver a high radiation dose to tumor lesion but a low dose to surrounding normal tissues, thereby achieving satisfactory efficacy and low toxicities. Numerous previous studies have reported the experience of radioactive Iodine-125 seed application in various cancer types including EC [13, 16, 17, 25–27]. Generally, the efficacy was satisfactory and side reactions were acceptable. This evidence further strengthened the application of radioactive Iodine-125 seed implantation in clinical practice.

In our study, we employed multicenter cohorts to report the efficacy and toxicity of radioactive Iodine-125 seed implantation in EC patients with recurrent lymph nodes after radiotherapy. Our study achieved a tumor response rate of 69.9%, a 2-year LPFS rate of 23.0%, and an OS rate of 38.8%. Compared with the efficacy (55.6% for tumor response, 18% for 2-year local control, 22% for 2-year survival) in the study by Zhang et al. [28], the efficacy in our study was better. The main difference regarding treatment-related factors was that the D90 was higher in our study than that in the study by Zhang et al. (median: 130.3 Gy vs. 104 Gy). Another main cause contributing to this difference should be the higher percentage of primary N2-3 disease (52.8% vs. 21.4%) in the study by Zhang et al. [28]. Another report consisting of 16 patients demonstrated a 15-month control rate of 33.3% which was similar to that in our study (data not shown). Taken these results together, radioactive Iodine-125 seed implantation should be a promising treatment strategy in recurrent EC after previous radiotherapy failure.

When performing multivariate analysis to identify independent prognostic factors associated with radioactive Iodine-125 seed treatment, we found that prescribed radiation dose and D90 were not prognostic factors. One of the main reasons for this should be that seeds were only implanted into selective lymph nodes for palliative care, with no anti-tumor effects on local or metastatic lesions. Therefore, the radiation dose contributed less to the survival benefit. Of note, radioactive Iodine-125 seed implantation relieved recurrent lymph node-related symptoms such as pain and numbness in 61.6% of patients, further supporting the role of radioactive Iodine-125 seed implantation in palliative care of advanced cancers. As expected, the initial tumor stage, tumor lesion volume, and short-term efficacy were independent prognostic factors for overall survival.

Concerning toxicity, the grade 3–5 skin toxicity in our study was similar to that reported by Ji et al. [13], but a little higher than that in other studies [27, 28]. As shown in our result, a high D90 dose was associated with more frequent grade 1 or more toxicity. Possibly, the difference in toxicity profile should be attributed to more intensive radioactive Iodine-125 seed schedules in our study. To point out, the study by Gao et al. [27] only focused on mediastinal lymph nodes recurrence which would also result in less skin toxicity compared with cervical lymph nodes recurrence. Moreover, their sample sizes were very small in these two studies (n = 36 and n = 16, respectively). Therefore, the toxicity profile in the two studies should be interpreted discreetly because. Notably, our study together with the previous study [13] both reported that the prescribed and D90 doses were not correlated with survival outcomes, which reminded us of a balance between radioactive Iodine-125 seed efficacy and toxicity.

The limitations of our work should also be addressed. The retrospective nature may subject our study to potential bias. Moreover, we only collected skin toxicity related to radioactive Iodine-125 seed implantation. Other adverse data should also be needed for better evaluation and interpretation of radioactive Iodine-125 seed implantation. In light of the technique used, some patients received only CT-guided radioactive Iodine-125 seed implantation but not the 3D-PCT or 3D-PNCT which have better accuracy. Notably, the overall survival data should be interpreted carefully since radioactive Iodine-125 seed implantation only affects the control of recurrent lymph nodes but not local and metastatic disease. A further head-to-head study is needed to evaluate the effect of radioactive Iodine-125 seed implantation on overall survival.

## Conclusion

Radioactive Iodine-125 seed implantation seems efficient with acceptable toxicity for the treatment of lymph node recurrence secondary to esophageal cancer. A head-to-head study is needed to further evaluate the survival benefit.

## Data Availability

The datasets used and/or analyzed during the current study are available from the corresponding author on reasonable request.
